# Investigation the therapeutic effect of mindfulness-based cognitive-behavioral counseling in pregnant women with body image dissatisfaction: A randomized controlled trial

**DOI:** 10.1016/j.eurox.2025.100387

**Published:** 2025-04-11

**Authors:** Mehnoosh Farahabadi, Abbas Amanollahi, Bahman Cheraghian, Zahra Abbaspoor

**Affiliations:** a Student Research Committee, Faculty of Nursing and Midwifery, Ahvaz Jundishapur University of Medical Sciences, Ahvaz, Iran; b Department of Counselling, School of Education and Psychology, Shahid Chamran University of Ahvaz, Ahvaz, Iran; c Department of Biostatistics and Epidemiology, School of Public Health, Ahvaz Jundishapur University of Medical Sciences, Ahvaz, Iran; d Department of Midwifery, Reproductive Health Promotion Research Center, Ahvaz Jundishapur University of Medical Sciences, Ahvaz, Iran

**Keywords:** Mindfulness-based cognitive behavioral therapy, Body image, Concerns, Pregnant women

## Abstract

**Background and aims:**

Dissatisfaction with body image during pregnancy is a common issue in the general population and can pose risks for both the mother and the child. The aim of this study was to investigate the therapeutic effect of mindfulness-based cognitive-behavioral counseling in pregnant women with body image dissatisfaction: A randomized controlled trial.

**Methods:**

In this study, 50 pregnant women who were over 26 weeks pregnant were randomly assigned to intervention and control groups. In the intervention group, Mindfulness-based cognitive behavioral therapy was taught to pregnant women over 8 consecutive sessions, while the control group received no intervention. Body image concerns questionnaire was completed by both groups before and after the intervention, and data were analyzed using descriptive statistics and multivariate analysis of covariance.

**Results:**

The results showed that before the intervention, the mean and standard deviation of body image concern scores in the intervention group was 58.36 ± 3.13 and in the control group, was 58.08 ± 3.31, but after the end of the intervention, in the intervention group it decreased to 41.40 ± 2.85 (p < 0.001), while in the control group, it decreased (57.64 ± 3.42) slightly (P = 0.951). The total scores for body image concern in the intervention group were significantly lower compared to the control group (p < 0.001).

**Conclusion:**

Mindfulness-based cognitive behavioral therapy can help reduce body image concerns in pregnant women. Therefore, it is recommended that mental health professionals utilize this approach to improve the psychological and physical well-being of pregnant women and assist them in modifying their attitudes towards body image.

## 1 Introduction

Body image refers to the mental representation that individuals hold of their physical appearance and condition [1]. Body image encompasses both conscious and unconscious beliefs and feelings about one’s body [2]. It reflects how a person perceives themselves and how they believe others perceive them [3].

Body image comprises three dimensions: perception, behavior, and attitude. Perception involves an individual’s judgment of their weight, size, and specific body features [1]. Behavior includes actions taken to alter one’s appearance and avoidance of situations where one might feel scrutinized. Attitude encompasses the thoughts, beliefs, and evaluations one holds regarding their body [4].

The congruence of an individual’s mental representation of their body with reality is a significant factor in mental health [5] and can have biological, psychological, and social consequences [6]. Currently, dissatisfaction with body image, defined as the negative mental evaluation of one’s body, is increasingly prevalent. Prior research has established a direct association between body dissatisfaction and eating disorders, low self-esteem, and depression [7], [8].

The physical body undergoes changes across various states of health, illness, and injury. These changes are not limited to specific conditions such as skin diseases, obesity, burns, and alopecia, but also occur in response to physiological transitions related to puberty, menopause, pregnancy, and even menstruation [9]. Body image dissatisfaction is a common concern among females, particularly pregnant women [10]. Body image dissatisfaction during pregnancy is considerably more prevalent than before pregnancy [11]. Studies indicate that the global prevalence of body image dissatisfaction ranges from 11 % to 72 % among pregnant women, with variations influenced by factors such as pre-pregnancy BMI and cultural context [12]. In the reviewed articles, body dissatisfaction has been reported between 30 % and 75 % in Iran [13]. Pregnancy is a sensitive period in a woman’s life marked by changes in both mind and body. Consequently, women experience alterations in their body shape, weight, and body image during pregnancy [14]. Pregnancy-related body changes often conflict with societal beauty standards, potentially exacerbating body image dissatisfaction[15].

Body image dissatisfaction during pregnancy is a concern because it poses risks to both the mother and the fetus [16], potentially leading to detrimental behaviors such as inappropriate dieting and excessive exercise. These behaviors can result in insufficient weight gain, preterm birth, low birth weight, and delayed child and maternal development [1]. Previous studies have shown that body image dissatisfaction during pregnancy is associated with increased anxiety and depression, as well as lower self-esteem in the mother [17], [18]. Another study found a significant negative correlation between body image concerns and quality of life among pregnant women in Iran [19]. A randomized controlled trial conducted in eastern Turkey demonstrated that mindfulness-based sexual counseling (MBSC) significantly reduced body image concerns among pregnant women experiencing sexual distress. The study reported that mean scores for body image concerns were significantly lower in the mindfulness group compared to the control group, indicating a reduction in body image concerns following the intervention [20]. Given that pregnancy is an inherent part of women’s lives, concerns about body image are intrinsic to the experience, and their unmanaged nature can adversely affect the physical and mental health of both mother and fetus. Therefore, identifying and addressing body image dissatisfaction during pregnancy is crucial. Various therapeutic approaches have been employed to address body image concerns in different populations, including cognitive restructuring therapy, reciprocal behavior analysis training, cognitive-behavioral group therapy, integrative spirituality therapy training, positive thinking resilience training, self-compassion training, positive psychology therapy, and dialectical behavior therapy [17]. Furthermore, methods such as aerobic exercise, Pilates, and hip-hop resistance training have also been utilized [21]. Among therapeutic approaches, traditional cognitive-behavioral therapies incorporating mindfulness have been used to address body image concerns in various groups. Mindfulness-based cognitive therapy is a brief, structured intervention based on Kabat-Zinn’s stress reduction model, integrating cognitive principles. The aim of teaching mindfulness through traditional cognitive therapy is not to alter the content of thoughts but to foster a different attitude or relationship with thoughts, emotions, and feelings, emphasizing sustained attention, present moment awareness, and a positive mindset [16]. de Jong et al. found that MBCT increased certain dimensions of body awareness, such as self-regulation and not distracting, which are related to how individuals perceive and manage their bodily sensations [22]. A study on Iranian women with breast cancer explored the effects of mindfulness-integrated cognitive behavior therapy on body image. The results showed a positive impact on body image, among other psychological outcomes, suggesting that mindfulness-based interventions can improve body image concerns in this population [23]. Considering the high prevalence of body image dissatisfaction during pregnancy and its significant impact on maternal mental health, and consequently on the health of the newborn and her family, coupled with the limited research on the effect of mindfulness-based cognitive behavioral therapy on body image dissatisfaction in pregnant women in Iran, we decided to undertake this study.

## 2 Materials and methods

The present study was a randomized clinical trial conducted on 50 pregnant women who were over 26 week’s gestation. The protocol of study was approved by the Ethics Committee of Ahvaz Jundishapur University of Medical Sciences (Ref. ID: IR.AJUMS.REC.1400.459). Also, it registered in the Iranian Registry of Clinical Trials (Ref. ID: IRCT20211218053448N1). Inclusion criteria included: being married; primiparous; gestational age over 26 weeks; obtaining a score greater than 48 from the Body Image Concerns Questionnaire (BICQ); individuals aged 20–40 years; and possessing at least a basic education. Exclusion criteria included: drug use or hospitalization due to mental illness in the past year; major stressors (serious illness of self or spouse or uncertain and worrying fetal position); death of a close relative; migration; accident; severe family disturbances in the past six months; abortion or stillbirth; desire for abortion, simultaneous participation in other psychotherapy sessions, and any previous cosmetic surgery. Written informed consent was given by all women prior to data collection.

### 2.1 Setting

The lead researcher visited two public health centers in Ahvaz city and screened pregnant women based on the inclusion/exclusion criteria. A total of 74 women were examined, of whom 50 who met the inclusion criteria were randomly assigned into experimental and control groups. Primipiparous women who attended to Public Health Centers No. 1 of East, Abuzar, and West in Ahvaz, during the winter of 2022. Women who met inclusion criteria and received a score more than 48 from Body Image Concerns Questionnaire were requested to fill-out the demographic questionnaire.

### 2.2 Randomization

The method of assigning women to the intervention or control groups was based on block randomization with a block size of 4 and allocation ratio of 1:1. The randomization list was prepared by a statistician. The code assigned to the eligible women were placed in sealed envelopes by someone who was not involved in the study and was not aware of the research objectives. The envelops were then given to the head of health centers. In this way, neither the researcher nor the participants were aware of group allocation until the commencement of the study. This study was single blind, and the researcher was aware of which group received the intervention. Also, because the counseling was done in a group setting, blinding was not possible, but participants were not aware of grouping.

### 2.3 Sample size

In order to determine the sample size, based on the methods of a previous study [24], and the following formula for comparing two means was used, α= 0.01, β= 0.10, X1 = 7.94, X2 = 1.74, S1 = 4.52, and S2= 4.47 were considered.

$$n=\frac{{\left(4^{2}+4.47^{2}\right)}^{2}{\left(1.96+1.32\right)}^{2}}{{\left(7.94-\overset{¯}{1.74}\right)}^{2}}=16$$

The initial sample size was calculated to be 32 individuals (16 in each group) and considering a maximum dropout rate of 35 % (due to the fact that pregnant women may be less likely to visit healthcare centers during the COVID-19 epidemic), the final sample size was determined to be 50 individuals (25 in each group).

### 2.4 Intervention

The study employed a quasi-experimental design, comprising two groups: an experimental group and a control group. The research incorporated both pre-test and post-test assessments. The independent variable in this study was the administration of mindfulness-based cognitive therapy (MBCT), which was exclusively applied to the experimental group. The investigation focused on the impact of MBCT on the post-test scores of the experimental group, in comparison to the control group. The dependent variables included factors such as body image, age, pregnancy-related issues, educational attainment, employment status, pregnancy status, and complications associated with pregnancy. These variables were carefully controlled and homogenized within each group to ensure comparability.

The participants in the experimental group underwent eight sessions of mindfulness-based cognitive-behavioral therapy. These sessions were held consistently on the same day each week for a duration of eight weeks, with each session lasting 45 minutes in the counseling room of the health center as a group. In order to learn and master the principles of MBCT, the lead researcher participated in a workshop held by an expert in MBCT. And after each session, telephone follow-up was done to remember the exercises at home. In contrast, the control group did not receive any educational or counseling interventions. Following the eighth session, participants from both groups were once again asked to complete a questionnaire assessing concerns about body image and individuals in the control group were provided with a CD containing educational material. The specifics of the session content are delineated in Table 1.

**Table 1:** Content of training sessions.

The objectives of sessions	The title of the sessions	NO
1- Introducing the method of mindfulness. 2- Determining the objectives of the session. 3- Presenting the title of the meeting and introducing the topic. 4- Setting the general policy considering the aspect of confidentiality. 5- Inviting the participants to form groups of two and introduce themselves to each other. 6- Practicing eating raisins. 7- Giving feedback and discussion about eating practice. 8- Awareness of thoughts and automatic guidance. 9- Awareness of thoughts and feelings and communication with them and emphasis on their impermanence. 10- Physical examination exercise. 11- Explanation about the homework based on the physical examination tape for one week. 12- Explanation about the presence of mind from daily activities. 13- Distribution of tapes and pamphlets of the first session. 14- Finish the class by focusing on short breathing.	Automatic guidance	Session 1
1- Practicing physical examination and emphasizing familiarity with body whispers. 2- Reviewing practice and giving feedback. 3- Reviewing last week's homework. 4-Practicing thoughts and feelings. 5- Recording the pleasant events. 6- Sitting meditation for 40–45 minutes. 7- Distribution of pamphlets of the second session among the participants. 8- Presentation of homework. 9- Physical examination 6 times in 7 days. 10- Breathing with the presence of mind for 45 or 40 minutes, 6 times in 7 days. 11- Recording of events Pleasant or enjoyable (every day). 12- Presence of mind from normal activities.	facing the obstacles	Session 2
1- Practice seeing or hearing. 2- Sitting meditation for 30–40 minutes. 3- Review exercise. 4- Review assignment. 5- Practicing breathing space for 3 minutes and reviewing. 6- Lying down with presence of mind. 7- Walking with the presence of mind and revision. 8- Presenting a list of unpleasant events. 9- Distribution of pamphlets of the third session. 10- Specify homework.	Knowing how the mind can often be busy and scattered, we learn to deliberately focusing awareness on breathing allows us to be more focused and integrated.	Session 3
1-Five-minute practice of seeing or hearing. 2- Forty-minute meditation. Awareness of breathing, body, voice and thoughts. 3- Review exercise. 4- Revision of homework. 5- eight-minute breathing space + review and reading a relaxing poem) in the version by Segal et al., it is the poetry of wild geese, which will be presented in this research based on Iranian and Islamic culture). 6-Copies of “the disaster of living pamphlets” for class. 7- Distributing pamphlets to the participants in the fourth session. 8- Determining homework.	Mostly, when the mind wants to focus on one subject and avoid other subjects, it gets distracted.	Session 4
1- Forty minutes of sitting meditation. 2- Connecting with reactions and experiences. 3- Review exercise. 4- Revision of home exercise. 5- Breathing space and its revision 6. Reading a poem by Rumi. 7- An 8-minute breathing space for confronting and revising. 8- Distribution of pamphlets of the fifth session among the participants. 9- Homework assignments.	In the presence of mind approach to be present at the same time, a person must look at events from a different angle to have a broad and different view of them.	Session 5
1- Sitting meditation for 40 minutes. 2- Revision of exercise. 3- Revision of homework 4. Preparing to finish the course. 5- Creation, thoughts and practice of points of view or surrogate thoughts. 6- Breathing time, and its review. 7- Distribution of pamphlets 8. Determination of homework.	Different communication means allowing yourself to be present to the experience, exactly as it is, without judging it or trying to change it from what it is.	Session 6
1–40 minutes sitting meditation. 2- Review exercises. 3- Revision of homework. 4- Practice observing the relationship between activity and mood. 5- Preparing a list of enjoyable activities and activities that lead to a sense of accomplishment 6. Planning and preparing a suitable program for such activities. 7–8-minute breathing space. 8- Identifying symptoms of relapse of depression and anxiety. 9- Identifying the work needed to face relapse/return. 10- Space for minute breathing or walking with the presence of mind. 11- Distribution of pamphlets of the sixth session among the participants. 12- Giving homework.	Negative moods and thoughts, limit our connection to experience. It is reasonable to understand that thoughts are just thoughts, even for someone who does not believe this.	Session 6
1- Body check exercise. 2- Revision of home exercise. 3- Revision of the entire program. 4- Distribution of the questionnaire among the participants. 5- Discussion on how to best practice the mobility and discipline that have been in the last 7 weeks. Whether to continue in regular or irregular exercises created. 6- Examining and discussing plans and finding positive reasons to continue practicing. 7- Distributing session 8 pamphlets among the participants. 8- Ending the classes with the last meditation.	Exercises to prevent the possibility of recurrence of depression and unpleasant states of anxiety	Session 8

Upon the completion of each session, participants were assigned to practice the learned techniques at home as homework. The prescribed number of repetitions for each technique was established by the researcher, adhering to the lecture protocol outlined by Segal et al. [25].

### 2.5 Outcomes

Assessments of Body Image Concerns, conducted at baseline and again at the 8-week mark following the intervention, demonstrated a significant effect attributable to the therapeutic intervention.

### 2.6 Instruments

#### 2.6.1 Socio-demographic questionnaire

This instrument encompasses a range of queries pertaining to demographic and health-related factors. It solicits information on the participant’s age, their spouse’s age, body mass index (BMI), educational attainment for both the participant and their spouse, usage of anti-anxiety medications, instances of psychiatric hospitalization, pregnancy-associated conditions (such as diabetes and hypertension), gestational age, occupation, residential setting (urban vs. rural), the intentionality of the pregnancy (desired vs. undesired), and any complications experienced during pregnancy.

#### 2.6.2 Body image concerns questionnaire (BICQ)

Developed by Littleton et al. [19], the BICQ is a 19-item measure that evaluates individuals’ concerns regarding their body image. Respondents indicate the frequency of their concerns using a five-point Likert scale ranging from 1 (never) to 5 (always). The questionnaire is divided into two subscales: one assessing dissatisfaction and embarrassment related to appearance (encompassing items 14, 9, 8, 5, 3, 15, 16, 17, 18, 19), and the other evaluating the impairment in personal functioning attributable to appearance concerns (including items 4, 6, 7, 10, 11, 12, 13). Scores can vary from 19 to 95, with higher scores denoting more pronounced negative body image perceptions, and lower scores reflecting a more positive self-image. In the Iranian context, Basaknejad and Ghaffari have affirmed the test’s reliability, reporting a Cronbach’s alpha coefficient of 0.95, in Iran [26].

### 2.7 Statistical analysis

In both the pre-test and post-test phases, descriptive statistics such as the mean and standard deviation were employed. The assumption of normality for the research variables across groups was evaluated using the Kolmogorov-Smirnov test. The normality assumption of the research variables by groups showed that Body dissatisfaction, Individual performance and Concern about body image in pregnant women (total) all variables are normal under both groups (P < 0.05). Data analysis encompassed the use of descriptive statistics, and depending on the nature of the data, either an independent *t*-test or a non-parametric Mann-Whitney *U* test was applied to investigate the associations between qualitative and quantitative variables within the treatment groups. To adjust for potential confounders, an analysis of covariance (ANCOVA) was utilized. All statistical analyses were conducted using SPSS software, version 24. A significance threshold of p < 0.05 was established for all tests.

## 3 Results

**Fig. 1:**
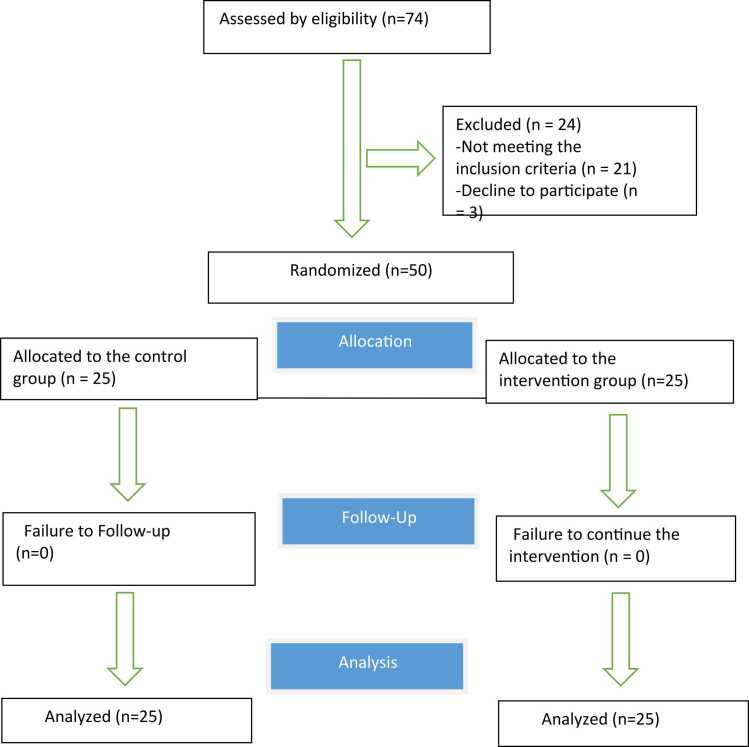
Flow-diagram of enrolling and retaining participants for the study.

Data were collected and analyzed from 50 pregnant women. All participants remained in the study until its completion (Fig. 1). The mean age, with standard deviation (SD), for the intervention group was 25.20 (1.27) years, and for the control group, it was 25.32 (3.88) years. The majority of women in both groups were employed and held a bachelor’s degree. According to the results in Table 2, there was no statistically significant difference between the two groups in terms of demographic factors (P < 0.05).

**Table 2:** The mean differences in socio-demographic variables by groups.

**Variable**		**Intervention Mean ± SD/ N (%)**	**Control Mean ± SD / N (%)**	P-Value*
**Age (years)**		25.20 ± 1.27	25.32 ± 3.88	0.911
**Spouse's age (years)**		29.24 ± 3.65	28.92 ± 3.65	0.758
**Gestational age (week)**		27.86 ± 0.56	27.72 ± 0.46	0.317
**BMI**		25.11 ± 1.16	25.20 ± 1.27	0.799
**Education**	Diploma	7 (70.0)	3(30.0)	0.519**
	Post-diploma	6 (50.0)	6 (50.0)	
	Bachelor	10 (41.7)	14 (58.3)	
	Master degree	2 (50.0)	2 (50.0)	
**Job**	Housewife	7 (43.8)	9 (56.2)	0.762**
	Employed	18 (52.9)	16 (47.1)	

* Chi-square test

** Independent T test

As shown in Table 3; according to the results of the independent *t*-test, the difference in the mean of dissatisfaction with appearance in the control and intervention groups is significant only after the intervention (p < 0.001). The mean of the variable of dissatisfaction with appearance after the intervention has decreased from 33.36 ± 2.37–23.92 ± 1.99. According to the results of the paired *t*-test, the difference in the mean before and after the intervention in the intervention group is significant (p < 0.001).

**Table 3:** Comparison the mean and SD of body image concerns and its sub scales before and after the intervention by groups.

	**Intervention Mean ± SD**	Control P-Value*Mean ± SD	P-Value*
**Dissatisfaction with the appearance**			
Before intervention After intervention P-value**	33.36 ± 2.37 23.92 ± 1.99 > 0.001	33.32 ± 2.23 33.20 ± 2.29 0.083	0.951 > 0.001
**Poor Individual performance**			
Before intervention After intervention degrees of freedom P-value**	25.00 ± 1.95 17.48 ± 1.42 18.04 (24) > 0.001	24.76 ± 2.02 24.44 ± 1.98 87.20 (24) 0.08	0.672 > 0.001
**Body image concern(total)**			
Before intervention After intervention degrees of freedom P-value**	58.36 ± 3.13 41.40 ± 2.85 22.63 (24) > 0.001	58.08 ± 3.31 57.64 ± 3.42 09.3 (24) 0.951	0.760 > 0.001

* Independent T test

** paired T test

Also, in relation to poor individual performance; the results of the independent *t*-test showed that the difference in the mean of the control and intervention groups was significant only after the intervention (p < 0.001). According to the results of the paired *t*-test, the difference in the mean before and after the intervention in the intervention group (p < 0.001) and control (p = 0.008) was significant. The mean of the variable of poor individual performance after the intervention in the control group decreased slightly from 24.76 ± 2.02–24.44 ± 1.98, while in the intervention group it decreased dramatically from 25.00 ± 1.95–17.48 ± 1.42.

Finally, regarding concerns about pregnant women's body image, according to the results of the independent *t*-test, the difference in the mean of the control and intervention groups is significant only after the intervention (p < 0.001). According to the results of the paired *t*-test, the difference in the mean before and after the intervention in the intervention group is significant (p < 0.001). The mean of the variable of concerns about pregnant women's body image (total) has decreased from 58.36 ± 3.13–41.40 ± 2.85 after the intervention.

## 4 Discussion

In this study, the researchers aimed to investigate the effect of Mindfulness-based cognitive behavioral therapy on pregnant women’s body image. The results indicated that after intervention, there was a significant difference in the total score of body image concern, as well as all measured dimensions, between the experimental and control groups.

Despite the absence of research specifically addressing the impact of Mindfulness-based cognitive behavioral therapy on the body image of pregnant women, evidence from studies conducted on other populations suggests that mindfulness counseling can have a beneficial effect on body image. For instance, Feizizadeh et al. [27] observed that mindfulness-based cognitive therapy contributed to a reduction in negative body image and the fear of negative evaluation among women with breast cancer post-mastectomy. Similarly, Katibaei et al. [28] reported a significant improvement in body image and self-esteem among adolescent girls, which was attributed to the cultivation of positive body imagery following cognitive reconstruction training. Given this significant impact, it can be concluded that a cognitive-behavioral mindfulness-based group intervention significantly reduced body image concern in pregnant women.

Moreover, Mosavi et al. [29] found that mindfulness-based cognitive therapy was effective in improving body image among male bodybuilders, utilizing meditation techniques. Lewer et al. (2017) conducted research on overweight women diagnosed with Binge Eating Disorder (BED) and found that cognitive-behavioral therapy led to a significant improvement in body image disorders [30].

In continuation, the results of this research are compared with the results of previous studies. In the study by Shahabi Zadeh and colleagues titled "The Effectiveness of Acceptance and Commitment Therapy and Mindfulness on Spousal Attachment, Dysfunctional Cognitive Processes (Worry and Rumination), and Social Body Anxiety in Primiparous Women in the Third Trimester of Pregnancy," it was also shown that there was a significant difference in social body anxiety scores between the control group and the two treatment groups—mindfulness (P < 0.006) and acceptance and commitment therapy (P < 0.002). This indicated that these two therapies significantly reduced social body anxiety in primiparous women in their third trimester of pregnancy. However, no significant difference was observed between the mindfulness group and the acceptance and commitment therapy group in social body anxiety scores (P = 0.32) [31]. Additionally, in a study by Naghdian et al. in 2020 titled "The Effectiveness of Compassion-Focused Therapy on Reducing Body Image Concern and Quality of Life in Breast Cancer Patients," it can be concluded that compassion-focused therapy is an effective strategy for helping breast cancer patients suffering from body image concern and depression due to its high efficiency, especially when conducted in groups, as it is cost-effective and well-received by patients [32]. Studies have shown that MBCT can effectively reduce symptoms of depression and anxiety in pregnant women, which are often linked to body image concerns[33], [34]. By addressing these mental health issues, MBCT may indirectly improve body image satisfaction. Also, MBCT enhances psychological well-being by promoting adaptive emotion regulation strategies and improving self-esteem[34]. These benefits can contribute to a more positive perception of one's body during pregnancy. Finally, MBCT offers a non-pharmacological intervention, which is particularly appealing during pregnancy when medication use is often limited. This makes it a safe and accessible option for managing body image concerns.

### 4.1 Strengths and limitations

This is the first study to evaluate the effect of mindfulness-based cognitive therapy on body image concerns of pregnant women, but it has some limitations. First, this study focused on pregnant women at 26 weeks' gestation or later, which may not be generalizable to studies of body image in pregnant women assessed in the first trimester or late pregnancy. Second, the study lacked comprehensive oversight regarding the participants’ adherence to prescribed home exercises, relying solely on self-reporting. Third, in this study, confounding factors such as history of cosmetic procedures or history of personality disorders were not considered. Lastly, the inherent individual and social differences, as well as the personal characteristics of the women, introduced variables that were beyond the control of the researchers, potentially influencing the responses to the questionnaires. Therefore, it is recommended that future studies be conducted with a larger sample size, in different trimesters, with more monitoring, and taking into account the personality and social characteristics of individuals.

## 5 Conclusion

It can be concluded that cognitive-behavioral strategies with a mindfulness approach significantly reduces the level of concern about body image among pregnant women. Consequently, the results of this research encourage gynecologists and midwives to pay more attention to the psychological aspects of their pregnant patients. Such attention could potentially contribute to improved management and overall pregnancy experiences. Given the positive effect of counseling on body image concerns and also considering that pregnant women are an important and influential group in society, considering health-oriented education based on group or individual counseling can pave the way for mental health reforms for this group. Thus, the results of the present study can be considered a path for health organizers. Also, by using counseling, we can provide specialized support in the clinical field and counseling to the pregnant women, in order to prevent their future problems. It is hereby suggested that mindfulness-based cognitive behavioral therapy be included as part of childbirth preparation classes and as part of prenatal care.

## Ethical considerations

This paper reports the findings of the research study that adhered to the Declaration of Helsinki.

This study received ethical approval from the Ethics Committee of Ahvaz Jundishapur University of Medical Sciences, under the ID IR.AJUMS.REC.1400.549. Additionally, it was registered with the clinical trial code IRCT20211218053448N1.

## Funding

This research was financially supported by Ahvaz Jundishapur University of Medical Sciences Ahvaz.

## CRediT authorship contribution statement

**Cheraghian Bahman:** Writing – review & editing, Formal analysis, Data curation. **Abbaspoor Zahra:** Writing – review & editing, Validation, Supervision, Project administration, Methodology, Investigation, Conceptualization. **Farahabadi Mehnoosh:** Writing – original draft, Resources, Project administration, Funding acquisition, Formal analysis. **Amanelahi Abbas:** Supervision, Investigation.

## Declaration of Competing Interest

The authors declare that this research was funded by Ahvaz Jundishapur University of Medical Sciences,.” The authors have no competing interests to declare.
